# Factors Regulating the Relationship Between Total and Size-Fractionated Chlorophyll-*a* in Coastal Waters of the Red Sea

**DOI:** 10.3389/fmicb.2019.01964

**Published:** 2019-09-09

**Authors:** Robert J. W. Brewin, Xosé Anxelu G. Morán, Dionysios E. Raitsos, John A. Gittings, Maria Ll. Calleja, Miguel Viegas, Mohd I. Ansari, Najwa Al-Otaibi, Tamara M. Huete-Stauffer, Ibrahim Hoteit

**Affiliations:** ^1^College of Life and Environmental Sciences, University of Exeter, Cornwall, United Kingdom; ^2^National Centre for Earth Observation, Plymouth Marine Laboratory, Plymouth, United Kingdom; ^3^Division of Biological and Environmental Sciences and Engineering, Red Sea Research Center, King Abdullah University for Science and Technology, Thuwal, Saudi Arabia; ^4^Department of Biology, National and Kapodistrian University of Athens, Athens, Greece; ^5^Department of Earth Science and Engineering, King Abdullah University of Science and Technology, Thuwal, Saudi Arabia; ^6^Department of Climate Geochemistry, Max Planck Institute for Chemistry, Mainz, Germany

**Keywords:** phytoplankton, size, chlorophyll, Red Sea, temperature

## Abstract

Phytoplankton biomass and size structure are recognized as key ecological indicators. With the aim to quantify the relationship between these two ecological indicators in tropical waters and understand controlling factors, we analyzed the total chlorophyll-*a* concentration, a measure of phytoplankton biomass, and its partitioning into three size classes of phytoplankton, using a series of observations collected at coastal sites in the central Red Sea. Over a period of 4 years, measurements of flow cytometry, size-fractionated chlorophyll-*a* concentration, and physical-chemical variables were collected near Thuwal in Saudi Arabia. We fitted a three-component model to the size-fractionated chlorophyll-*a* data to quantify the relationship between total chlorophyll and that in three size classes of phytoplankton [pico- (<2 μm), nano- (2–20 μm) and micro-phytoplankton (>20 μm)]. The model has an advantage over other more empirical methods in that its parameters are interpretable, expressed as the maximum chlorophyll-*a* concentration of small phytoplankton (pico- and combined pico-nanophytoplankton, $C_{p}^{m}$ and $C_{p,n}^{m}$, respectively) and the fractional contribution of these two size classes to total chlorophyll-*a* as it tends to zero (*D*_*p*_ and *D*_*p,n*_). Residuals between the model and the data (model minus data) were compared with a range of other environmental variables available in the dataset. Residuals in pico- and combined pico-nanophytoplankton fractions of total chlorophyll-*a* were significantly correlated with water temperature (positively) and picoeukaryote cell number (negatively). We conducted a running fit of the model with increasing temperature and found a negative relationship between temperature and parameters $C_{p}^{m}$ and $C_{p,n}^{m}$ and a positive relationship between temperature and parameters *D*_*p*_ and *D*_*p,n*_. By harnessing the relative red fluorescence of the flow cytometric data, we show that picoeukaryotes, which are higher in cell number in winter (cold) than summer (warm), contain higher chlorophyll per cell than other picophytoplankton and are slightly larger in size, possibly explaining the temperature shift in model parameters, though further evidence is needed to substantiate this finding. Our results emphasize the importance of knowing the water temperature and taxonomic composition of phytoplankton within each size class when understanding their relative contribution to total chlorophyll. Furthermore, our results have implications for the development of algorithms for inferring size-fractionated chlorophyll from satellite data, and for how the partitioning of total chlorophyll into the three size classes may change in a future ocean.

## 1. Introduction

Phytoplankton are a critical component of the Earth's system. Absorbing incoming solar radiation, CO_2_ and synthesizing organic matter, they are responsible for half of the planetary primary production (Longhurst et al., 1995; Field et al., 1998), modulate oceanic carbon, and provide energy for the majority of marine life. Light absorption by phytoplankton in the ocean is dependent on its biomass. Most of the light absorbed by phytoplankton is lost as heat, thus variations in phytoplankton biomass modulate solar heating in the ocean (Sathyendranath et al., 1991). A small component of absorbed light is used by phytoplankton in photosynthesis, making phytoplankton biomass critical for marine primary production and for energy transfer to higher trophic levels, impacting global fisheries catch (Chassot et al., 2010).

A second important characteristic of phytoplankton is its size structure. A suite of phytoplankton biochemical functions are controlled by size, including: metabolic rate, growth and nutrient uptake (Platt and Jassby, 1976; Platt and Denman, 1977, 1978; Maloney and Field, 1991; Chisholm, 1992; Marañón, 2009, 2015; Finkel et al., 2010). The absorption of light by an assemblage of phytoplankton of known biomass varies with size structure (Morel and Bricaud, 1981; Prieur and Sathyendranath, 1981; Bricaud et al., 2004; Devred et al., 2006; Brewin et al., 2011). Therefore, phytoplankton size also influences photosynthetic rate and ocean heating (Sathyendranath and Platt, 2007; Uitz et al., 2008; Brewin et al., 2017b). The sinking rates of phytoplankton are impacted by size, with large-celled phytoplankton thought to be responsible for a large fraction of export production and small-celled phytoplankton for recycled production (Eppley and Peterson, 1979; Michaels and Silver, 1988; Boyd and Newton, 1999; Laws et al., 2000; Guidi et al., 2009; Briggs et al., 2011; Mouw et al., 2016), at the same time acknowledging small-celled phytoplankton carbon export can also be significant (Mouw et al., 2016; Richardson, 2019). The size of phytoplankton is also thought to influence the structure of the marine food chain (Legendre and Le Fevre, 1991; Maloney and Field, 1991). These are some of the reasons why phytoplankton biomass and size structure are considered as two key ecological indicators in the marine environment (Platt and Sathyendranath, 2008).

A common measure of phytoplankton biomass is the total chlorophyll-*a* concentration (representing the sum of mono- and divinyl chlorophyll-*a*, chlorophyllide-a, and the allomeric and epimeric forms of chlorophyll-*a*, hereafter referred to collectively as total chlorophyll), the major photosynthetic pigment in marine phytoplankton. Unlike phytoplankton carbon, which is more difficult to measure, total chlorophyll can be routinely estimated *in situ* (e.g., fluorometrically or using High Performance Liquid Chromatography, HPLC) or through satellite remote-sensing of ocean color (O'Reilly et al., 1998). Conventionally, phytoplankton size structure is quantified by partitioning biomass (total chlorophyll) into three size classes [pico- (<2 μm), nano- (2–20 μm) and micro-phytoplankton (>20 μm); Sieburth et al., 1978], with the role of each thought to differ in the cycling of key elements such as carbon, with taxonomic composition, nutrient concentrations and environmental conditions influencing the composition of the three size classes (IOCCG, 2014).

The relationship between total chlorophyll and that contained in each of the three size classes has been studied thoroughly in some regions (Raimbault et al., 1988; Chisholm, 1992; Goericke, 2011; Marañón et al., 2012; López-Urrutia and Morán, 2015), with picophytoplankton known to contribute most to total chlorophyll at low concentrations, nanophytoplankton at intermediate concentrations, and microphytoplankton at high concentrations (IOCCG, 2014). This relationship has been quantified statistically (Uitz et al., 2006), empirically (Hirata et al., 2011) and more mechanistically (Brewin et al., 2010; Devred et al., 2011), at local and global scales (IOCCG, 2014). One approach to modeling this relationship, that has proven to be a popular choice (e.g., Brotas et al., 2013; Lin et al., 2014; Sammartino et al., 2015; Sahay et al., 2017; Hu et al., 2018; Lamont et al., 2018; Liu et al., 2018; Sun et al., 2018), is the three-component model of Brewin et al. (2010). The model is based on two exponential functions (Sathyendranath et al., 2001; Devred et al., 2006) that relate the fraction of total chlorophyll by combined pico- and nanophytoplankton (*F*_*p,n*_, cells <20 μm) and picophytoplankton (*F*_*p*_, cells <2 μm) to total chlorophyll concentration (*C*) according to

(1) $$\begin{array}{l} F_{p,n}=\frac{C_{p,n}^{m}\left[1-\exp\left(-\frac{D_{p,n}}{C_{p,n}^{m}}C\right)\right]}{C}, \end{array}$$

and

(2) $$\begin{array}{l} F_{p}=\frac{C_{p}^{m}\left[1-\exp\left(-\frac{D_{p}}{C_{p}^{m}}C\right)\right]}{C}. \end{array}$$

Model parameters are relatively easy to interpret, with $C_{p,n}^{m}$ and $C_{p}^{m}$ representing the asymptotic maximum chlorophyll concentrations for the associated size classes (<20 μm and <2 μm, respectively), and *D*_*p,n*_ and *D*_*p*_ representing the fraction of each size-class relative to total chlorophyll as total chlorophyll tends to zero. Once suitable parameters are obtained, and *F*_*p,n*_ and *F*_*p*_ derived, the fractions of nano- (*F*_*n*_) and micro-phytoplankton (*F*_*m*_) can be computed as *F*_*n*_ = *F*_*p,n*_ − *F*_*p*_ and *F*_*m*_ = 1 − *F*_*p,n*_. The chlorophyll concentration in each size class (*C*_*p*_, *C*_*n*_, and *C*_*m*_) can be calculated simply by multiplying the fractions (*F*_*p*_, *F*_*n*_, and *F*_*m*_) by total chlorophyll (*C*). Table 1 defines variables, parameters and abbreviations used in the manuscript.

**Table 1:** Symbols and definitions.

**Symbol**	**Definition**
*C*	Total chlorophyll concentration (mg m^−3^)
*C*_*p*_	Chlorophyll concentration for picoplankton (cells < 2μm) (mg m^−3^)
*C*_*p,n*_	Chlorophyll concentration for combined nano-picoplankton (cells < 20μm) (mg m^−3^)
*C*_*n*_	Chlorophyll concentration for nanoplankton (cells 2 − 20μm) (mg m^−3^)
*C*_*m*_	Chlorophyll concentration for microplankton (cells > 20μm) (mg m^−3^)
$C_{p,n}^{m}$	Asymptotic maximum value of *C*_*p,n*_ (cells < 20 μm) (mg m^−3^)
$C_{p}^{m}$	Asymptotic maximum value of *C*_*p*_ (cells < 2 μm) (mg m^−3^)
DOC	Dissolved organic carbon (μmol L^−1^)
*D*_*p,n*_	Fraction of total chlorophyll in combined nano-picoplankton (cells < 20μm) as total chlorophyll tends to zero
*D*_*p*_	Fraction of total chlorophyll in picoplankton (cells < 2μm) as total chlorophyll tends to zero
*F*_*p*_	Fraction of total chlorophyll for picoplankton (cells < 2μm)
*F*_*p,n*_	Fraction of total chlorophyll for combined nano- picoplankton (cells < 20μm)
*F*_*n*_	Fraction of total chlorophyll for nanoplankton (cells 2 − 20μm)
*F*_*m*_	Fraction of total chlorophyll for microplankton (cells > 20μm)
*G*_1_	Parameter of Equation (5) controlling lower and/or upper bound in $C_{p,n}^{m}$
*G*_2_	Parameter of Equation (5) controlling slope of change in $C_{p,n}^{m}$ with *T*
*G*_3_	Parameter of Equation (5) controlling the *T* mid-point of *G*_2_
*G*_4_	Parameter of Equation (5) controlling lower and/or upper bound in $C_{p,n}^{m}$
*H*_1_	Parameter of Equation (6) controlling lower and/or upper bound in $C_{p}^{m}$
*H*_2_	Parameter of Equation (6) controlling slope of change in $C_{p}^{m}$ with *T*
*H*_3_	Parameter of Equation (6) controlling the *T* mid-point of *H*_2_
*H*_4_	Parameter of Equation (6) controlling lower and/or upper bound in $C_{p}^{m}$
*J*_1_	Parameter of Equation (7) controlling lower and/or upper bound in *D*_*p,n*_
*J*_2_	Parameter of Equation (7) controlling slope of change in *D*_*p,n*_ with *T*
*J*_3_	Parameter of Equation (7) controlling the *T* mid-point of *J*_2_
*J*_4_	Parameter of Equation (7) controlling lower and/or upper bound in *D*_*p,n*_
*K*_1_	Parameter of Equation (8) controlling lower and/or upper bound in *D*_*p*_
*K*_2_	Parameter of Equation (8) controlling slope of change in *D*_*p*_ with *T*
*K*_3_	Parameter of Equation (8) controlling the *T* mid-point of *K*_2_
*K*_4_	Parameter of Equation (8) controlling lower and/or upper bound in *D*_*p*_
MAD	Median absolute difference between estimated and measured data
*r*	Pearson correlation coefficient
RFU	Relative red fluorescence
RMSD	Root mean square difference between estimated and measured data
*T*	Water temperature (°C)
TDN	Total dissolved nitrogen (μmol L^−1^)

Although such models have proven successful at capturing the relationship between total chlorophyll and chlorophyll contained in each size class, it has been recognized that such relationships may be perturbed by climate variability (Brewin et al., 2012; Racault et al., 2014; Agirbas et al., 2015), potentially impacting how the marine ecosystem functions (Sathyendranath et al., 2017). Furthermore, relationships have been shown to differ with changes in environmental conditions, for example, with changes in water temperature and light availability (Brewin et al., 2015b, 2017a; Ward, 2015). To predict the response of the marine ecosystem to fluctuations in climate, it is critical to improve our understanding of how the relationships between these two key ecological indicators may change with changing environmental conditions. Among the warmest and most saline waters on the planet (Longhurst, 2007; Belkin, 2009; Raitsos et al., 2011; Yao et al., 2014a,b), and believed to reflect environmental conditions predicted in other marine regions decades from now (Christensen et al., 2007), the Red Sea is an interesting location to explore relationships between these indicators and environmental variability.

In this study, we make use of a dataset collected in coastal waters of the central Red Sea over a 4-year period, consisting of measurements of total chlorophyll, size-fractionated chlorophyll, picophytoplankton (abundance and cell properties by flow cytometry) and nutrient concentrations. We use these data, together with the three-component model of Brewin et al. (2010), with the aim to quantify the relationship between total and size-fractionated chlorophyll in tropical waters and improve our understanding on what controls this relationship. Specifically, we aim to address the following two research questions: (1) Is the relationship between total and size-fractionated chlorophyll in coastal waters of the Red Sea consistent with that observed in other ocean basins? (2) What factors influence the relationship between total and size-fractionated chlorophyll?

## 2. Methods

### 2.1. Study Area: Coastal Waters of the Red Sea

**Figure 1:**
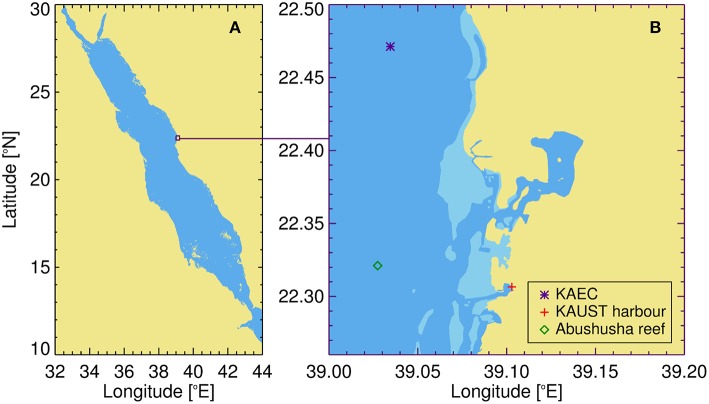
Study site. **(A)** Location of the study site with respect to the broader Red Sea. **(B)** Study site near the coastal waters of Thuwal in the Kingdom of Saudi Arabia and the locations of the three datasets used in the study. KAEC refers to the King Abdullah Economic City and KAUST to the King Abdullah University for Science and Technology.

The chosen study site was located in the central Red Sea (Figure 1A) in the coastal waters near Thuwal in the Kingdom of Saudi Arabia (Figure 1B). We made use of water samples collected by King Abdullah University for Science and Technology (KAUST) at three locations: (1) in KAUST harbor (22.3065°N, 39.1029°E; Silva et al., 2019), where weekly sampling of surface waters was conducted between 2015 and 2017 and monthly sampling of surface waters during 2018; (2) near King Abdullah Economic City (KAEC, 22.4712°N, 39.0345°E, ~700 m depth; Calleja et al., 2018), where surface waters (5 m depth) were sampled around midday covering the seasonal variability between 2015 and 2017, on board of KAUST R.V. Thuwal and KAUST R.V. Explorer; and (3) near Abushusha reef, just offshore from KAUST (22.321°N, 39.027°E; see Figure 1B), at the surface of a ~70 m depth station, sampled on a monthly basis during 2018 on board the KAUST Durrat Al-Bahr Almar 1 and 5 vessels. All water samples were collected during daylight hours (08:30–14:30 local time) using a pre-clean (acid-washed) polycarbonate 9 L carboy (KAUST Harbor and Abushusha reef) or Niskin bottles (the rest of the sampling).

### 2.2. Size-Fractionated Filtration (SFF) Data

The size-fractionated filtration (SFF) method for determining the chlorophyll concentration in each size class involves filtering water through filters of different pore sizes. For each water sample collected, 200 ml of sea water were filtered sequentially through 20, 2, and 0.2 μm polycarbonate filters. Following filtration, the filters were stored at −80°C for at least 24 h. Pigment extraction was made by submerging the filters in 90 % acetone for 24 h at 4°C. Samples were then analyzed using a Triology Fluorometer (Turner Designs), pre- and post-calibrated using pure chlorophyll-*a* as a standard (*Anacustis nidulans*, Sigma Aldrich). The total chlorophyll concentration was taken as the sum of the size fractions for each sample. The concentration of chlorophyll passing through the 2 μm filter and retained on the 0.2 μm filter was designated as picophytoplankton chlorophyll (*C*_*p*_), that passing through the 20 μm filter was designated as pico- and nano-phytoplankton chlorophyll (*C*_*p,n*_), the chlorophyll retained on the 20 μm filter was designated as microphytoplankton chlorophyll (*C*_*m*_), and the concentration of chlorophyll retained on the 2 μm filter, having passed through the 20 μm filter, was designated as nanophytoplankton chlorophyll (*C*_*n*_). The fractions of each size class relative to total chlorophyll (*F*_*p*_, *F*_*p,n*_, *F*_*n*_, and *F*_*m*_) were computed by dividing the chlorophyll concentration in each size class (*C*_*p*_, *C*_*p,n*_, *C*_*n*_, and *C*_*m*_) by total chlorophyll concentration (*C*). In total, 136 SFF samples were available, 8 from KAEC, 116 from KAUST harbor and 12 from Abushusha reef.

### 2.3. Model Parameterization

Model parameters ($C_{p,n}^{m}$, $C_{p}^{m}$, *D*_*p,n*_, and *D*_*p*_) for the three-component model of Brewin et al. (2010) were derived by fitting Equations (1) and (2) using a standard, nonlinear least-squared fitting procedure (Levenberg-Marquardt, IDL Routine MPFITFUN, Moré, 1978; Markwardt, 2008) using the *F*_*p*_, *F*_*p,n*_ and *C* SFF data as input. The parameters *D*_*p,n*_ and *D*_*p*_ were constrained to be less than or equal to one, since size-fractionated chlorophyll cannot exceed total chlorophyll. The method of bootstrapping (Efron, 1979; Brewin et al., 2015b) was used to randomly resample (utilizing IDL Routine RANDOMU) with replacement the dataset and re-fit equations for each iteration (1,000 iterations). Median values and 95% confidence intervals were taken from the resulting parameter distributions (see Table 2). Model parameters are compared with other model fits using SFF data in other ocean basins (Table 2).

**Table 2:** Parameter values for Equations (1) and (2) compared with parameters derived using the size-fractionated filtration (SFF) method in other regions.

**Study**	**Parameters for Equations (1) and (2)**				**Location**	***N*^#^**
	${C_{p,n}}^{m^{*}}$	${C_{p}}^{m^{*}}$	***D*_*p,n*_**	***D*_*p*_**		
This Study^*$*^	1.23 (0.83↔2.78)	0.43 (0.33↔0.68)	0.94 (0.86↔1.0)	0.66 (0.58↔0.73)	Red Sea	136
Brewin et al., 2014b^*$*^	2.61 (1.82↔4.09)	0.73 (0.54↔1.11)	0.95 (0.92↔0.98)	0.76 (0.71↔0.82)	Atlantic Ocean	408
Corredor-Acosta et al., 2018^*$*^	2.12 (1.75↔2.54)	0.19 (0.11↔0.27)	0.92 (0.88↔0.96)	0.21 (0.16↔0.33)	Central-southern Chile	182
Ward, 2015	0.79	0.16	0.98	0.85	Global Ocean	620

$ *Model parameters are computed as the median of the bootstrap parameter distribution and bracket parameter values refer to the 2.5% and 97.5% confidence intervals on the distribution*.

# *N, Number of samples used for model parameterization*.

* *denotes units in mg m^−3^*.

### 2.4. Flow Cytometry, Nutrient Sampling and Physical Variables

For the 136 samples with SFF data, measurements of flow cytometry, nutrients, dissolved organic carbon (DOC) and total dissolved nitrogen (TDN) were also collected. The abundances of three picophytoplankton groups, *Prochlorococcus, Synechococcus* and picoeukaryotes, were obtained from each water sample using BD FACSCanto flow cytometer, applying the methodology as detailed in Gasol and Morán (2015). We measured the red fluorescence as a proxy for the chlorophyll content and the right angle light scatter or side scatter (SSC) as a proxy of cell size, following Calvo-Díaz and Morán (2006). These values were made relative to those of the 1 μm latex fluorescent beads added to each sample as internal standard (Molecular Probes, ref. F-13081). The empirical calibration between relative SSC and cell diameter described in Calvo-Díaz and Morán (2006) was used to estimate the cell size of each of the three picophytoplankton groups.

Nutrients were measured by filtering seawater through pre-combusted (450 °C, 4.5 h) GF/F filters. The samples were subsequently frozen and stored at −20°C until analysis. Nitrate, nitrite, silicate, and phosphate were analyzed using a segmented flow analyzer from Seal Analytical, with standards prepared in acid-washed glassware using a nutrient-free artificial seawater matrix (Silva et al., 2019). Samples for DOC and TDN analysis were passed through an online acid-cleaned polycarbonate filter cartridge, holding a pre-combusted (450 °C, 4.5 h) GF/F filter, attached directly to the Niskin bottle, and collected into acid cleaned and pre-combusted glass vials. Samples were acidified with H_3_PO_4_ until a pH of 1-2, and kept in the dark at 4°C until analysis at the laboratory by high temperature catalytic oxidation (HTCO) using a Shimadzu TOC-L (Calleja et al., 2019). The accuracy of the estimates were monitored using reference material of deep-sea carbon water (42–45 μmol C L^−1^ and 31–33 μmol N L^−1^) and low carbon water (1–2 μmol C L^−1^) provided by D. A. Hansell (Univ. of Miami).

Water temperature and salinity measurements were collected for each sample. In KAUST harbor and at Abushusha reef, this was conducted immediately prior to sampling with an environmental probe (YSI probe; Silva et al., 2019). At KAEC, water temperature and salinity measurements were obtained using a SBE 9 (Sea-Bird Electronics) Conductivity-Temperature-Depth (CTD) probe. All data used in this study can be accessed in the Supplementary Material.

### 2.5. Statistical Tests

To evaluate the model performance, the Pearson linear correlation coefficient (*r*, IDL Routine CORRELATE) and the median absolute difference (MAD) were used. The significance (*p*) of the correlation coefficient (*r*) was computed using the t-statistic and applying a two-sided *t*-test (utilizing IDL Routine T_PDF). The correlation was deemed significant if *p* < 0.05 and highly significant if *p* < 0.001. The MAD was computed as

(3) $$\begin{array}{l} \text{MAD}=\text{median}\left(\left|X_{i,E}-X_{i,M}\right|\right), \end{array}$$

where *X* is the variable, subscript *i* denotes the index in the data series, from 1 to *N* where *N* is the length of the series, the subscript *M* denotes the measured variable and *E* the estimated variable from the model. Considering that the chlorophyll concentration is approximately log-normally distributed (Campbell, 1995), statistical tests were performed in log_10_ space when using chlorophyll as the variable (unless explicitly stated), and in linear space when using the fraction of total chlorophyll in each size class as the variable. The MAD was used as it is robust to non-Gaussian distributions and outliers. For comparison with results from other studies, we also computed the root mean square difference (RMSD), according to

(4) $$\text{RMSD}={\left[\frac{1}{N}\sum_{i=1}^{N}{\left(X_{i,E}-X_{i,M}\right)}^{2}\right]}^{1/2}.$$

## 3. Results and Discussion

### 3.1. Fit of Three-Component Model to SFF Data

**Figure 2:**
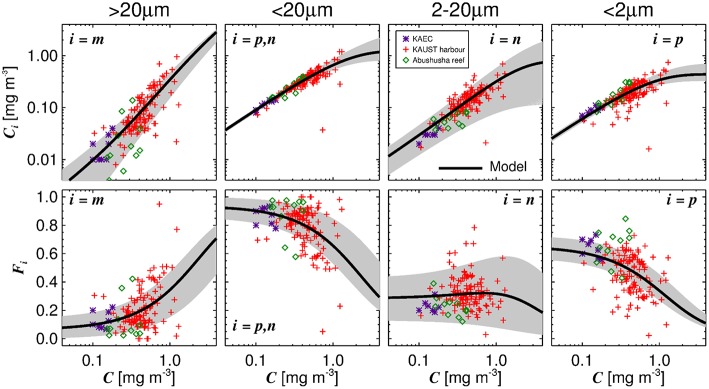
Fits of the three-component model to size-fractionated filtration (SFF) data collected in the study. Top row shows the absolute chlorophyll concentrations (*C*_*m*_, *C*_*p,n*_, *C*_*n*_, and *C*_*p*_) and bottom row the fractions (*F*_*m*_, *F*_*p,n*_, *F*_*n*_, and *F*_*p*_) plotted as a function of total chlorophyll (*C*), with the tuned three-component model (parameters from Table 2) overlain. Gray shading represents a model ensemble varying parameters between their confidence intervals (Table 2).

The three-component model was seen to capture the general changes in size-fractionated chlorophyll (*C*_*p*_, *C*_*n*_, *C*_*p,n*_, and *C*_*m*_) and fractions of total chlorophyll (*F*_*p*_, *F*_*n*_, *F*_*p,n*_, and *F*_*m*_) when plotted as a function of total chlorophyll (Figure 2 and Table 3). Statistical performance indicates that the three-component model fits the SFF data well (Table 3), with comparable or lower RMSD values when compared with model fits in other regions using SFF measurements. Model parameters also compare favorably with other model fits using SFF data in other ocean basins (Table 2). The conceptual framework of the three-component model is seen to hold in coastal Red Sea waters, with the abundance of small cells increasing to a given chlorophyll concentration, beyond which chlorophyll increases through the addition of larger size classes of phytoplankton (Raimbault et al., 1988; Chisholm, 1992; Goericke, 2011). This upper bound for small cells increases with increasing size (Brewin et al., 2014b), with assemblages of phytoplankton <20 μm in size having a significantly higher upper bound ($C_{p,n}^{m}$) than assemblages of phytoplankton <2 μm in size ($C_{p}^{m}$, see Table 2). In agreement with other studies (IOCCG, 2014), picophytoplankton contribution to total chlorophyll is highest at low total chlorophyll, nanophytoplankton at intermediate total chlorophyll, and microphytoplankton at high total chlorophyll (Figure 2).

**Table 3:** Performance of the three-component model fit to the Red Sea data and a comparison with fits of the model in other regions using size-fractionated filtration (SFF) data.

**Variable**	**This study** **Red Sea**			Brewin et al. (2014b) **Atlantic Ocean**			Ward (2015)^*****#*****^ **Global dataset**			Corredor-Acosta et al. (2018) **Central-southern Chile**			**This study (temperature-dependent)** **Red Sea**		
	***r***	**MAD**	**RMSD**	***r***	**MAD**	**RMSD**	***r***	**MAD**	**RMSD**	***r***	**MAD**	**RMSD**	***r***	**MAD**	**RMSD**
*C*_*p*_ ^*^	0.66	0.09	0.18	0.86	–	0.20	0.46	–	0.43	0.37	–	0.42	0.67	0.08	0.18
*C*_*p,n*_ ^*^	0.82	0.05	0.13	0.97	–	0.09	0.93	–	0.12	0.81	–	0.20	0.83	0.04	0.12
*C*_*n*_ ^*^	0.80	0.11	0.17	–	–	–	0.88	–	0.30	0.80	–	0.22	0.79	0.09	0.17
*C*_*m*_ ^*^	0.75	0.18	0.30	–	–	–	0.91	–	0.47	0.88	–	0.41	0.80	0.16	0.27
*F*_*p*_	0.43	0.10	0.13	–	–	–	–	–	–	–	–	–	0.47	0.09	0.14
*F*_*p,n*_	0.41	0.08	0.14	–	–	–	–	–	–	–	–	–	0.58	0.07	0.12
*F*_*n*_	0.11	0.07	0.12	–	–	–	–	–	–	–	–	–	0.07	0.06	0.12
*F*_*m*_	0.41	0.08	0.14	–	–	–	–	–	–	–	–	–	0.58	0.07	0.12

* *Statistical tests performed in log_10_ space*.

# *From the temperature independent model of Ward (2015)*.

### 3.2. Relationship Between Model Residuals and Other Variables

Although the model fits the data reasonably well, it is by no means perfect (Table 3, Figure 2). Differences between the model and data can be related either to uncertainties in the measurements (Brewin et al., 2014a), or simply to inability of the model to account for real variability surrounding the general relationship between size-fractionated chlorophyll and total chlorophyll.

Whereas the SFF method has an advantage in that the sizes of phytoplankton are explicitly partitioned, in comparison with other methods of determining size-fractionated chlorophyll (e.g., by High Performance Liquid Chromatography pigment analysis; Vidussi et al., 2001; Uitz et al., 2006; Brewin et al., 2010; Devred et al., 2011; Kheireddine et al., 2017), there are still uncertainties in the measurements. The filters can retain particles smaller than the nominal pore size, which is dependent on the morphology of the particles, cohesiveness of the particles, volume filtered and on the filter types used (Sheldon, 1972; Logan, 1993; Logan et al., 1994; Chavez et al., 1995; Gasol and Morán, 1999; Knefelkamp et al., 2007; Dall'Olmo et al., 2009). On the other hand, a certain portion of particles larger than the nominal pore size can also pass through the filter (e.g., from overlapping holes), and be accounted for in smaller-size fractions. This is dependent on whether the phytoplankton break apart during the filtration process, on the morphology of the particles, and on their orientation as they pass through the filter. The impact of these factors on measurement uncertainties is difficult to quantify, though it has been suggested that the clogging of filters and the inability to define accurately the pore size of filters, are two key issues (Droppo, 2000). Simultaneous measurements made by multiple types of *in situ* methods are needed to make an accurate diagnosis of uncertainty in the SFF technique (Nair et al., 2008; Brewin et al., 2014a). Though beyond the scope of this study, future efforts are needed in this direction.

While acknowledging that the measurements have uncertainties, to explore how the relationship between total and size-fractionated chlorophyll could be influenced by other ecological factors and consequently how the three-component model could be improved, we investigated whether the residuals (model minus measurement) were correlated with other variables in the dataset. We focused on the differences between model and measurement for *F*_*p,n*_ and *F*_*p*_, considering that these fractions were used to parametrize the model (Equations 1 and 2).

Table 4 shows correlations between residuals in *F*_*p,n*_ and *F*_*p*_ and other variables in the dataset. As anticipated, there is no correlation between residuals and total chlorophyll, highlighting that the model fit captured the variation in *F*_*p,n*_ and *F*_*p*_ as a function of total chlorophyll. For *F*_*p,n*_, highly significant (*p* < 0.001) correlations were observed with temperature (positive), and significant correlations (*p* < 0.05) with picoeukaryote cell abundance (negative) and salinity (positive). For *F*_*p*_, significant (*p* < 0.05) correlations were observed with temperature, TDN, silicate, nitrite (all positive) and picoeukaryote cell abundance (negative). Of all the variables, *F*_*p,n*_ and *F*_*p*_ were both significantly correlated with temperature and picoeukaryote cell number. These two variables were inversely correlated (*r* = −0.40, *p* < 0.001) in the dataset, with higher picoeukaryote cell numbers in the winter and lower picoeukaryote cell numbers in summer.

**Table 4:** Correlations between model residuals (model minus measurements) in the fraction of total chlorophyll by combined pico- and nano-phytoplankton (*F*_*p,n*_) and picophytoplankton (*F*_*p*_, cells <2 μm) and other variables collected in the dataset.

**Variable**	*******F*******_*********p**, **n*********_			*******F*******_*********p*********_		
	***r***	***p***	***N***	***r***	***p***	***N***
Total chlorophyll (*C*)	0.00	0.962	136	−0.03	0.740	136
Temperature	**0.34**	**0.000**	**134**	**0.26**	**0.002**	**134**
Salinity	**0.23**	**0.008**	**133**	−0.07	0.398	133
DOC	0.16	0.073	132	0.03	0.737	132
TDN	0.16	0.072	133	**0.17**	**0.045**	**133**
Silicate	0.05	0.590	122	**0.18**	**0.046**	**122**
Nitrite	0.12	0.193	122	**0.26**	**0.004**	**122**
Nitrate	0.06	0.533	122	0.15	0.090	122
Phosphate	−0.09	0.322	121	0.07	0.464	121
Picoeukaryotes cells ^*^	**–0.18**	**0.040**	**131**	**–0.20**	**0.022**	**131**
Synechococcus cells ^*^	−0.04	0.638	131	−0.13	0.127	131
Prochlorococcus cells ^*^	−0.26	0.187	27	−0.25	0.212	27

*Bold indicates significant correlations (*p* < 0.05)*.

* *Cell numbers were log_10_ transformed when running the correlations*.

**Figure 3:**
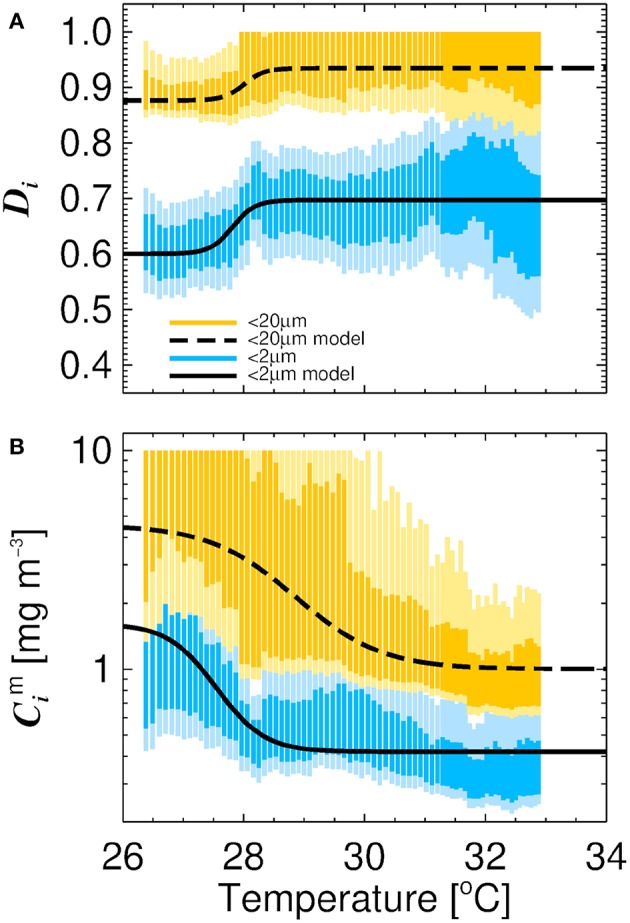
Relationship between the parameters of the three-component model and water temperature derived from sorting the dataset and conducted a running fit of the model (bin size 60 measurements) with increasing temperature. Average water temperature of each bin is on the abscissa and 13.6 and 86.4 % (darker shading) and 2.5 % and 97.5 % (lighter shading) confidence intervals of the parameters from a bootstrap fit (1,000 iterations) on the ordinate (confidence intervals are constrained to realistic values, 0 to 10 for $C_{p,n}^{m}$ and $C_{p}^{m}$ and <1 for *D*_*p,n*_ and *D*_*p*_). **(A)** Shows the relationship between temperature and the parameters *D*_*p,n*_ and *D*_*p*_. Solid black line is the model of Brewin et al. (2017b) tuned to the data (Equation 8) for cells <2 μm and dashed line for cells <20 μm (Equation 7). **(B)** Shows the relationship between temperature and the parameters $C_{p,n}^{m}$ and $C_{p}^{m}$. Solid black line is the model of Brewin et al. (2017b) tuned to the data (Equation 6) for cells <2 μm and dashed line (Equation 5) for cells <20 μm.

Residuals between the three-component model and fitted data have previously been shown to vary with temperature in polar waters and in the North Atlantic (Ward, 2015; Brewin et al., 2017a), but not in tropical seas with temperatures consistently exceeding 22 °C, suggesting seasonality may also play an important role in tropical waters. To investigate the impact of temperature on the parameters of the three-component model we followed a similar approach to Brewin et al. (2017a). This involved sorting the dataset by increasing temperature and conducted a running fit of the model (Equations 1 and 2) as a function of temperature using a bin size of 60 samples. This involved sliding the bin from low to high temperature and fitting Equations (1) and (2) each time the bin slides (increments of 1 sample). For each fit, we used the method of bootstrapping (1,000 iterations), and derived 13.6 and 86.4 % confidence intervals (1 standard deviation), as well as 2.5 and 97.5 % confidence intervals (2 standard deviation), for each parameter distribution in each bin (Figure 3), and assessed the relationship between the median parameters and average temperature of the bins.

We observed a positive relationship between *D*_*p,n*_ (fraction of cells <20 μm to *C* as *C* tends to zero) and temperature (*r* = 0.80, *p* < 0.001) and *D*_*p*_ (fraction of cells <2 μm to *C* as *C* tends to zero) and temperature (*r* = 0.63, *p* < 0.001, Figure 3), and an inverse relationship between $C_{p,n}^{m}$ and temperature (*r* = −0.51, *p* < 0.001) and $C_{p}^{m}$ and temperature (*r* = −0.89, *p* < 0.001). To capture these relationships, we fitted logistic functions to the data following the approach of Brewin et al. (2017a). The quantities $C_{p,n}^{m}$ and $C_{p}^{m}$ were modeled as functions of temperature (*T*) according to

(5) $$\begin{array}{l} C_{p,n}^{m}=1-\left\{\frac{G_{1}}{1+\exp\left[-G_{2}\left(T-G_{3}\right)\right]}+G_{4}\right\}, \end{array}$$

and

(6) $$\begin{array}{l} C_{p}^{m}=1-\left\{\frac{H_{1}}{1+\exp\left[-H_{2}\left(T-H_{3}\right)\right]}+H_{4}\right\}, \end{array}$$

where *G*_1_ and *G*_4_ represent the upper and lower bounds of $C_{p,n}^{m}$, *G*_2_ the slope of change in $C_{p,n}^{m}$ with *T*, and *G*_3_ the *T* mid-point of the slope between $C_{p,n}^{m}$ and *T*. For $C_{p}^{m}$, *H*_*i*_, where *i* = 1 to 4, is analogous to *G*_*i*_ for $C_{p,n}^{m}$. Similarly, *D*_*p,n*_ and *D*_*p*_ were modeled as a function of temperature (*T*) according to

(7) $$\begin{array}{l} D_{p,n}=\frac{J_{1}}{1+\exp\left[-J_{2}\left(T-J_{3}\right)\right]}+J_{4}, \end{array}$$

and

(8) $$\begin{array}{l} D_{p}=\frac{K_{1}}{1+\exp\left[-K_{2}\left(T-K_{3}\right)\right]}+K_{4}, \end{array}$$

where *J*_1_ and *J*_4_ represent the upper and lower bounds of *D*_*p,n*_, *J*_2_ the slope of change in *D*_*p,n*_ with respect to *T*, and *J*_3_ the *T* mid-point of the slope between *D*_*p,n*_ and *T*. For *D*_*p*_, *K*_*i*_ is analogous to *J*_*i*_ for *D*_*p,n*_. The parameters for Equations (5)–(8) were derived by fitting the equations to the median parameter values for each bin and average temperature of each bin, using a nonlinear least-squared fitting procedure with bootstrapping (utilizing IDL Routines described in Section 2.3), and by constraining input to plausible values (0 to 10 for $C_{p,n}^{m}$ and $C_{p}^{m}$ and <1 for *D*_*p,n*_ and *D*_*p*_). Parameter values for Equations (5)–(8) are provided in Table 5. The functions are seen to capture the general relationships observed in the dataset (Figure 3). Nonetheless, as this analysis is based on a relatively small dataset (136 samples), we recognize additional data is required to substantiate the relationship between model parameters and temperature observed here.

**Table 5:** Parameter values for Equations (5)–(8).

**Model parameter**	**Equation**	**Parameters for Equations (5) and (8)**^*******$*******^			
$C_{p,n}^{m}$ ^*^	5	*G*_1_ = –3.56 (±1.33)	*G*_2_ = –1.47 (±0.86)	*G*_3_ = 28.34 (±0.87)	*G*_4_ = 0.00 (±0.171)
$C_{p}^{m}$ ^*^	6	*H*_1_ = 1.20 (±0.30)	*H*_2_ = 2.58 (±2.23)	*H*_3_ = 27.28 (±0.60)	*H*_4_ = –0.61 (±0.58)
*D*_*p,n*_	7	*J*_1_ = 0.058 (±0.010)	*J*_2_ = 5.86 (±4.87)	*J*_3_ = 28.01 (±0.31)	*J*_4_ = 0.88 (±0.01)
*D*_*p*_	8	*K*_1_ = 0.097 (±0.019)	*K*_2_ = 5.34 (±4.49)	*K*_3_ = 27.82 (±0.21)	*K*_4_ = 0.60 (±0.02)

$ *Model parameters are computed as the median of the bootstrap parameter distribution and bracket parameter values refer to median absolute deviation on the distribution*.

* *Denotes units in mg m^−3^*.

**Figure 4:**
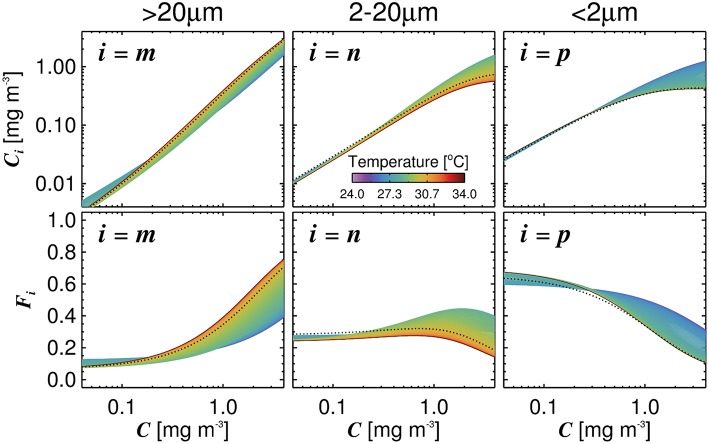
Influence of temperature on the relationship between size-fractionated chlorophyll and total chlorophyll, when incorporating Equations (5)–(8) into the three-component model. Top row shows the size-fractions of chlorophyll and bottom row the fractions of total chlorophyll in each size class, all plotted as a function of total chlorophyll. Dashed black lines refer to the model using a single set of parameters (Table 2).

After Equations (5)–(8) were incorporated into the model, residuals between the temperature-dependent model and data were no longer significantly correlated with water temperature or picoeukaryote cell number (*p* > 0.05 for both *F*_*p,n*_ and *F*_*p*_ for these correlations), confirming that the new parameterization accounted for the relationships originally observed between the residuals and model output (Table 4). Furthermore, model performance was seen to improve using the temperature-dependent model, with lower MAD values for all size classes and higher correlation coefficients and lower RMSD for most size classes (Table 3). Figure 4 illustrates how the relationship between size-fractionated chlorophyll and total chlorophyll changes with temperature, when incorporating Equations (5)–(8) into the model.

**Figure 5:**
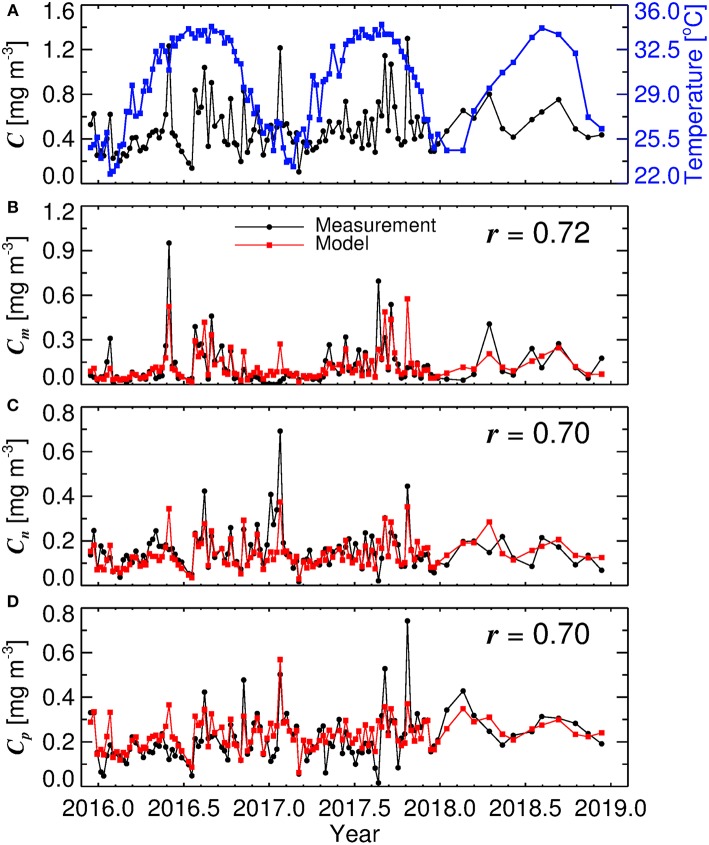
Time-series of data collected at KAUST harbor in Thuwal between 2016 and 2019. **(A)** time-series of water temperature and total chlorophyll (*C*), **(B)** microphytoplankton chlorophyll (*C*_*m*_), **(C)** nanophytoplankton chlorophyll (*C*_*n*_), and **(D)** picophytoplankton chlorophyll (*C*_*p*_). *r* represents the correlation coefficient between measurements and model (conducted in linear space).

Figure 5A shows a time-series of water temperature and total chlorophyll at KAUST harbor between 2016 and 2019. Clear seasonal cycles are seen in temperature, but not for total chlorophyll, with sporadic variations occurring at different times. Figures 5B–D show chlorophyll for micro-, nano- and picophytoplankton from *in situ* data (black) and estimates from the model (red), driven by total chlorophyll and water temperature (Figure 5A, Equations 1, 2, 5, 6, 7, and 8). In a highly-complex coastal environment, the three-component model is seen to explain around 50 % of the variance in size-fractionated chlorophyll (*r* ≥ 0.7 correlation in linear space, Figure 5). Considering that both water temperature (sea-surface temperature) and chlorophyll are accessible through satellite visible and thermal radiometry, the approach offers the potential for estimating size-fractionated chlorophyll from satellite data in the central Red Sea.

### 3.3. Influence of Changes in Taxonomic Composition of Picophytoplankton on Model Parameters

**Figure 6:**
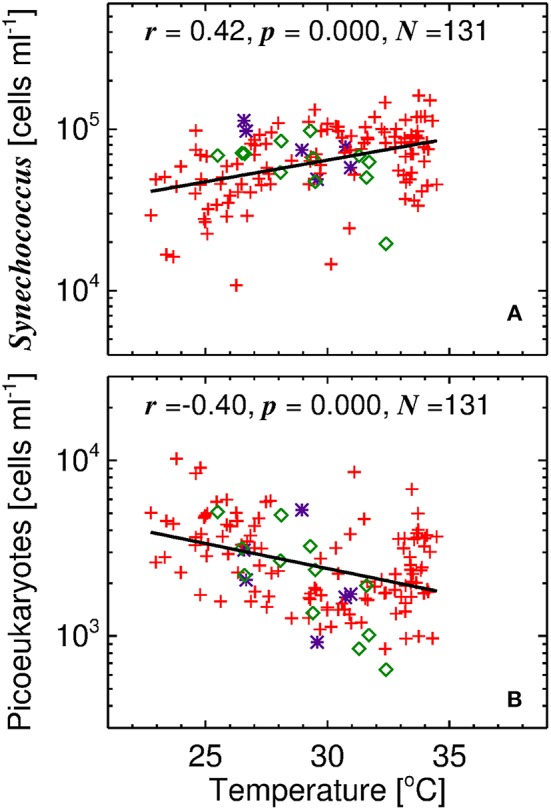
Relationship between temperature and picophytoplankton cell counts. **(A)** *Synechococcus* vs. temperature and **(B)** Picoeukaryotes vs. temperature. Solid line is a linear regression and symbols follow those of Figures 1, 2.

Our understanding of how model parameters change with temperature can be guided by analysing the flow cytometry data. Figure 6 shows the relationship between temperature and cell abundance for the two dominant picophytoplankton, *Synechococcus* and picoeukaryotes, as determined by flow cytometry. For the three sites sampled, with differing conditions (depth and picophytoplankton community composition), there is a clear shift in the composition of picophytoplankton with temperature, *Synechococcus* being positively correlated with temperature and picoeukaryotes inversely correlated (Figure 6).

Table 6 lists the average relative red fluorescence and cell size for each community of picophytoplankton derived from the flow cytometry data. Picoeukaryotes, as expected, were found to be larger in size and to have higher relative red fluorescence than the cyanobacteria (Table 6), consistent with studies in other regions (Blanchot et al., 2001; Calvo-Díaz et al., 2008). Relative red fluorescence has been used as a proxy of per cell chlorophyll concentration (Olson et al., 1983; Li et al., 1993; Veldhuis et al., 1997; Veldhuis and Kraay, 2000; Calvo-Díaz and Morán, 2006; Calvo-Díaz et al., 2008; Álvarez et al., 2017), acknowledging that there are natural variations in fluorescence per unit of chlorophyll among species (Sosik et al., 1989), size (Veldhuis et al., 1997), and with changes in phytoplankton physiology (Veldhuis and Kraay, 1993). Other factors can also impact fluorescence measured by a flow cytometer (Neale et al., 1989). If we multiply the relative red fluorescence for all picophytoplankton groups by their respective abundances, sum them up, then compare with *C*_*p*_ derived from SFF measurements, we obtain a reasonable positive correlation (*r* = 0.38, *p* < 0.001, *N* = 131), confirming the use of relative fluorescence as proxy of per cell chlorophyll concentration in our dataset. The increase in $C_{p}^{m}$ with decreasing temperature could therefore be associated with increasing picoeukaryotes numbers at lower temperature. This group of picophytoplankton is larger in size (1.31 μm for picoeukaryotes compared with 0.89 μm for *Synechococcus* and 0.76 μm for *Prochlorococcus*, see Table 6) and can store higher concentrations of chlorophyll per cell (Table 6), and may consequently result in higher $C_{p}^{m}$ values. Similarly, considering *C*_*p*_ constitutes the dominant portion of *C*_*p,n*_ in our dataset (Figure 2), that picoeukaryote red fluorescence was found to be correlated with *C*_*n*_ (*r* = 0.38, *p* < 0.001, *N* = 131) and *C*_*p,n*_ (*r* = 0.33, *p* < 0.001, *N* = 131), and that the presense of picoeukaryotes is often associated with the presense of larger nanoeukaryotes (Tarran et al., 2006; Tarran and Bruun, 2015), similarly links could be made with increases in the parameter $C_{p,n}^{m}$ at low temperature. Nonetheless, additional evidence (e.g., taxonomic composition of the larger size classes) is needed to substantiate these linkages.

**Table 6:** Average relative red fluorescence and cell size for each community of picophytoplankton derived from the flow cytometry data.

**Variable**	Picoeukaryotes ^*^ (*N* = 131)	Synechococcus^*^ (*N* = 131)	Prochlorococcus^*^ (*N* = 27)
Relative red fluorescence (RFU)	0.89 (±0.22)	0.031 (±0.008)	0.004 (±0.001)
Cell diameter (μm)	1.31 (±0.05)	0.89 (±0.02)	0.76 (±0.01)

* *Bracketed values ± represent the median absolute deviation of the data*.

With regards to parameters *D*_*p*_ and *D*_*p,n*_, it is worth recalling that these parameters reflect the fraction of each size-class relative to total chlorophyll as total chlorophyll tends to zero (i.e., ultra-oligotrophic waters). Picophytoplankton are thought to dominate in oligotrophic conditions, owing to their competitive advantage over larger cells in low nutrient conditions, a result that is consistent with our model parameterization over the entire temperature range (*D*_*p*_ > 0.6, Figure 4). However, we see marginally higher *D*_*p*_ and *D*_*p,n*_ parameters in warmer waters (summer, higher *Synechococcus* cell numbers) than cooler waters (winter, higher picoeukaryote cell numbers). A decrease in *D*_*p*_ and *D*_*p,n*_ with temperature has also been observed in other regions, over a different temperature range (Brewin et al., 2017a). There may be some direct effect of temperature on growth rates of the different picophytoplankton communities (Eppley, 1972; Chen et al., 2014) and their grazers (Steinberg and Landry, 2017), that cause these differences and allow for an increasing presence of larger cells (nano- and micro) in cooler oligotrophic waters. However, it is worth noting that, as most of the dataset is very coastal, chlorophyll concentrations rarely fall below 0.1 mg m^−3^ (Figure 2) making it difficult to interpret variations in *D*_*p*_ and *D*_*p,n*_ in this dataset. Future efforts to sample more oligotrophic regions of the Red Sea may shed further light on variations in these two parameters.

### 3.4. Understanding the Relationship Between Phytoplankton Biomass and Size Structure in a Future Ocean

Two key ecological indicators, phytoplankton biomass and size structure, are seen to covary in a predictable manner in coastal waters of the Red Sea (Figure 2), with small cells dominant at low chlorophyll concentrations and large cells at high concentrations, consistent with studies in other regions (Raimbault et al., 1988; Chisholm, 1992; Uitz et al., 2006; Brewin et al., 2010). These predictable relationships have been exploited for the development of ocean-color algorithms (IOCCG, 2014), and for the validation of, and assimilation of data into, marine ecosystem models (Ward et al., 2012; Hirata et al., 2013; Holt et al., 2014; de Mora et al., 2016; Ciavatta et al., 2018; Skákala et al., 2018). However, it has been recognized that such relationships might be perturbed by changes in climate (Sathyendranath et al., 2017).

The size-structure of the phytoplankton affects export of large aggregates (Boyd and Newton, 1999), with large cells thought to contribute more to the flux of carbon at depth than smaller phytoplankton, at similar levels of total chlorophyll (Guidi et al., 2009), acknowledging small-celled carbon export can be significant (Richardson, 2019). The photosynthetic rate of phytoplankton, for a given concentration of total chlorophyll, has been shown to depend on size-structure (Platt and Jassby, 1976; Fernández et al., 2003; Morán et al., 2004; Uitz et al., 2008; Álvarez et al., 2016; Brewin et al., 2017b; Curran et al., 2018; Robinson et al., 2018a,b). Biological heating by phytoplankton is influenced by the chlorophyll-specific absorption coefficient, which changes with size (Bricaud et al., 2004; Devred et al., 2006; Uitz et al., 2008; Brewin et al., 2011). The structure of the marine food web has also been found to depend on size composition of phytoplankton (Maloney and Field, 1991). Models that tie primary production and total chlorophyll, export production and total chlorophyll, predict energy flow and biological heating using total chlorophyll, are all vulnerable to shifts in the relationship between total and size-fractionated chlorophyll.

Standard, empirical algorithms used by space agencies for estimating total chlorophyll from blue-green reflectance ratios, derived from satellite measurements of ocean color, have been shown to incorporate implicitly a fixed relationship between size-fractionated chlorophyll and total chlorophyll (IOCCG, 2014), with low total chlorophyll concentrations represented by the optical properties of small cells and high concentrations by large cells (Dierssen, 2010; Sathyendranath et al., 2017). These algorithms are also vulnerable to shifts in the relationship between total and size-fractionated chlorophyll, with implications for using ocean-color data to detect climate variability (Sathyendranath et al., 2017). Tying the relationship between total and size-fractionated chlorophyll to other environmental factors (e.g., temperature) could aid in ocean-color algorithm development.

Results from this study indicate that, in the coastal waters of the Red Sea, changes in the taxonomic composition of the phytoplankton within a size class may affect the chlorophyll in that size class. Therefore, to predict future changes in size-fractionated chlorophyll, we need to understand how phytoplankton taxonomic composition is likely to change. In the coastal waters of the Red Sea, we found temperature to correlate with taxonomic composition of picophytoplankton and the partitioning of total chlorophyll into the three size classes. Other studies in the Red Sea, using different methods, have confirmed the influence of temperature on phytoplankton taxonomic composition (Pearman et al., 2017). Temperature has been shown as a key variable for predicting changes in taxonomic composition in tropical oceans (Flombaum et al., 2013; Lange et al., 2018; Agusti et al., 2019), temperate regions (Morán et al., 2010; Flombaum et al., 2013; Brewin et al., 2017a), and in polar waters (Li et al., 2009; Ward, 2015). Furthermore, as temperature is a variable that is routinely measured from space, its integration into models of ocean color could lead to improved estimates of size-fractionated chlorophyll (Raitsos et al., 2008; Ward, 2015; Brewin et al., 2017a), as well as other regional ocean-color products used in ecological studies (Brewin et al., 2013, 2015a; Raitsos et al., 2013, 2015, 2017; Racault et al., 2015; Gittings et al., 2018; Kheireddine et al., 2018), putting us in a better position to harness ocean-color data for detecting shifts in marine ecosystems in the Red Sea.

## 4. Summary

Using datasets of size-fractionated chlorophyll, flow cytometry, physical variables and nutrient concentrations, collected over a 4-year period in the coastal waters of the central Red Sea, we analyzed the relationship between total chlorophyll and its partitioning into three size classes of phytoplankton (pico-, nano- and micro-phytoplankton) to address two research questions: (1) Is the relationship between total and size-fractionated chlorophyll in coastal waters of the Red Sea consistent with that observed in other ocean basins? and (2) What factors influence the relationship between total and size-fractionated chlorophyll? A conceptual, three-component model was fitted to the data, that describes the relationship between total chlorophyll and those in the three size classes. Model fits and model parameters were comparable to studies fitting the model to datasets in other ocean basins, demonstrating, in answer to research question (1), that the relationship between total and size-fractionated chlorophyll in the coastal waters of the Red Sea is consistent with that observed in other ocean basins. We found the residuals in the fits to be significantly correlated with water temperature (positively) and picoeukaryote cell abundance (negatively), demonstrating, in answer to research question (2), that temperature and taxonomic composition are key factors influencing the relationship between total and size-fractionated chlorophyll in the coastal waters of the Red Sea.

We introduced a temperature-dependency on model parameters that was subsequently found to improve performance. Temperature was inversely related with picoeukaryote cell abundance, with higher picoeukaryote cell abundances in winter (cold) than summer (warm). Picoeukaryotes are known to contain higher chlorophyll per cell than picophytoplanktonic cyanobacteria and be larger in size, possibly explaining a decrease in the maximum chlorophyll concentration of small cells in the model ($C_{p}^{m}$ and $C_{p,n}^{m}$) with increasing temperature. This was supported by additional analysis using the relative red fluorescence and cell size estimates from flow cytometer data. However, we recognize additional evidence is needed to substantiate the link between the temperature dependence of model parameters and changes in the taxonomic composition of phytoplankton. Our results highlight the importance of temperature and taxonomic composition of phytoplankton within each size class when exploring the relationship between size-fractionated and total chlorophyll. This has implications for the development of satellite ocean-color algorithms and for predicting how ecosystem functioning may change in a future ocean.

## Data Availability

All datasets generated for this study are included in the manuscript and/or the Supplementary Material.

## Author Contributions

RB, XM, DR, JG, and IH proposed the study. XM, MC, MV, MA, NA-O, and TH-S collected the data. RB synthesized the data, re-tuned and further-developed the algorithms, organized, prepared and wrote the first version of the manuscript, and prepared all figures and tables. All authors contributed to the subsequent versions of the manuscript.

### Conflict of Interest Statement

The authors declare that the research was conducted in the absence of any commercial or financial relationships that could be construed as a potential conflict of interest.
